# Multiple calcaneal fibrous dysplasia: A case report

**DOI:** 10.1097/MD.0000000000018389

**Published:** 2019-12-20

**Authors:** Jong-Hyun Ko, Gun-Joo Park, Kwang-Bok Lee

**Affiliations:** Department of Orthopedic Surgery, Chonbuk National University Medical School, Research Institute of Clinical Medicine of Chonbuk National University-Biomedical Research Institute of Chonbuk National University Hospital, Jeonju, South Korea.

**Keywords:** calcaneus, fibrous dysplasia, monostotic

## Abstract

**Rationale:**

Fibrous dysplasia (FD) is a benign bone tumor due to developmental failure in the process of primitive bone remodeling to mature lamellar bone. The most common locations of monostotic FD of the extremity bones are the proximal femur, tibia, humerus and the radius. FD in the calcaneus is extremely rare and usually manifests clinically as a single bone lesion. Moreover, no research has reported on multiple lesions in calcaneal FD.

**Patient concerns:**

We report a 21-year-old man presented to our institution with pain upon walking for 2 months.

**Diagnoses:**

We diagnosed the patient with multiple calcaneal FD through histologic examination of the excised biopsy that revealed cellular, spindly stroma and woven bone without osteoblastic rimming resembling Chinese characters

**Interventions:**

Plain X-ray, computed tomography, magnetic resonance imaging and histologic examination. An excisional biopsy with extended curettage and bone grafting with allogenous bone and autogenous bone marrow aspirate concentrate were performed.

**Outcomes:**

No complications developed after surgery and during serial follow-ups at 3, 6 and 12 months. At a postoperative 12-month follow-up, a plain radiogram showed a well-consolidated bone graft in the lesions.

**Lessons:**

Calcaneal FD is rare disease entity. This case can help guide clinical decision-making in the future.

## 1 Introduction

Fibrous dysplasia (FD) is well-known as a benign bone tumor due to developmental failure in the process of primitive bone remodeling to mature lamellar bone that may involve 1 (monostotic) or more bones (polyostotic) in the body.^[1]^ The most common locations of monostotic FD of the extremity bones are the proximal femoral shaft, tibia, humerus and the radius.^[1]^

FD in the calcaneus is extremely rare and usually manifests as a single lesion in the bone. An extensive English literature search revealed only 3 published case reports on this topic.^[1–3]^ Moreover, there exist no research reports on multiple lesions in calcaneal FD. Thus, we provide the first case study on multiple calcaneal FD that can help guide clinical decision-making in the future.

### 1.1 Consent

The patient signed informed consent for the publication of this case report and any accompanying images. Ethical approval of this study was waived by the ethics committee of Chonbuk National University Hospital because it was a case report.

## 2 Case report

**Figure 1:**
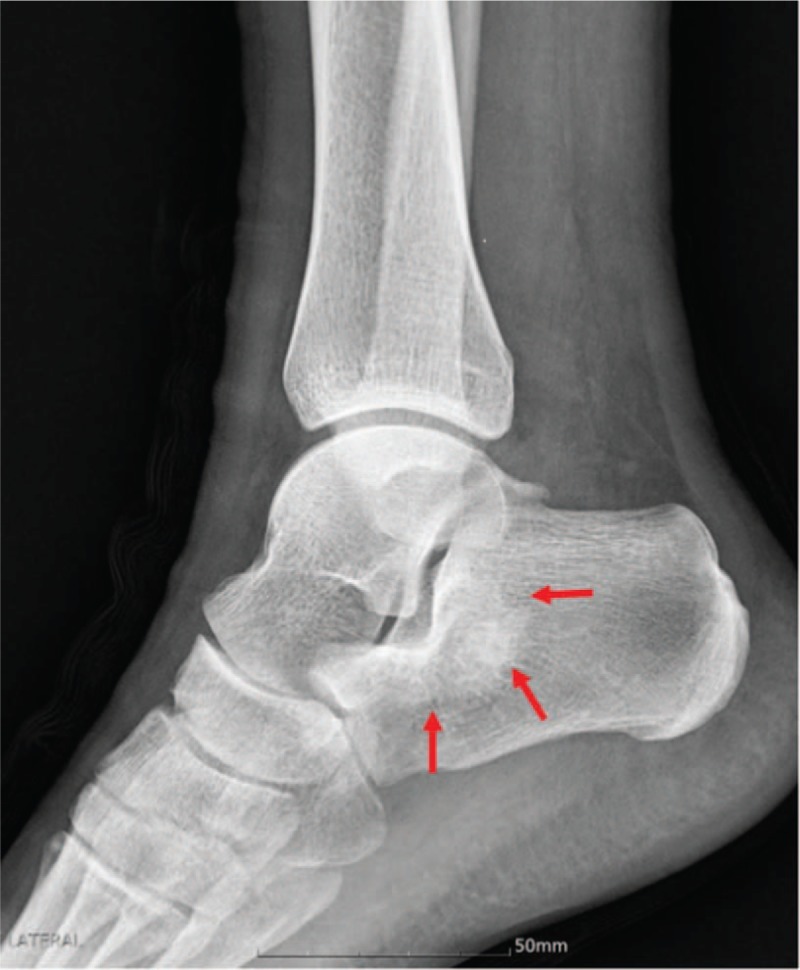
Preoperative plain radiogram shows an ill-defined osteoblastic lesion around the subtalar joint in the body of the calcaneus measuring 2.5 cm × 3.0 cm.

A 21-year-old man presented to our institution with pain upon walking for 2 months. No mass was palpated. There was no trauma and past medical history. On physical exam, there was no swelling or pain of the lateral aspect of the right ankle and hindfoot. The patient exhibited a full range of motion of his right ankle during the physical exam. A plain radiogram showed an ill-defined osteoblastic lesion around the subtalar joint in the body of the calcaneus measuring 2.5 cm × 3.0 cm (Fig. 1).

**Figure 2:**
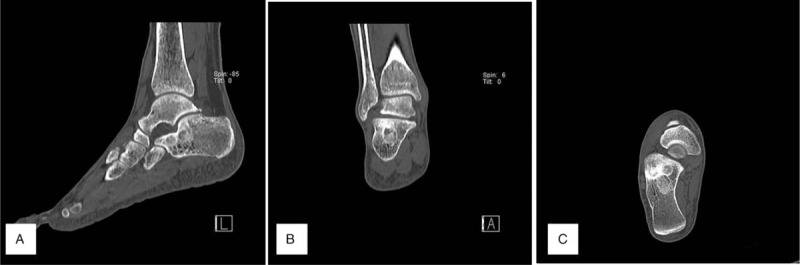
CT scan shows a sclerotic and osteoblastic lesion under the subtalar joint with a cystic round hole in the calcaneal body (A: sagittal, B:coronal, C: axial). CT = computed tomography.

**Figure 3:**
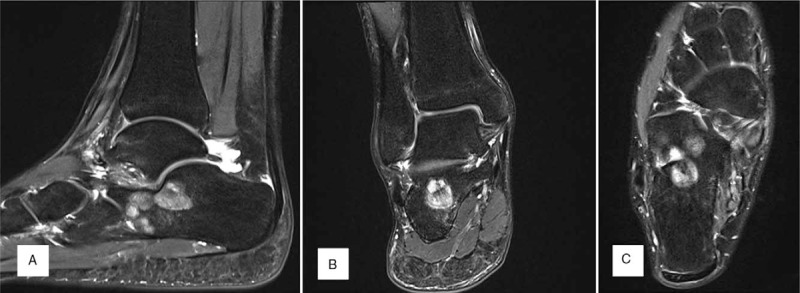
T2-weighted MR image shows 5 round-shaped lesions of high signal intensity with mixed low signal intensity (A: sagittal, B: coronal, C: axial).

**Figure 4:**
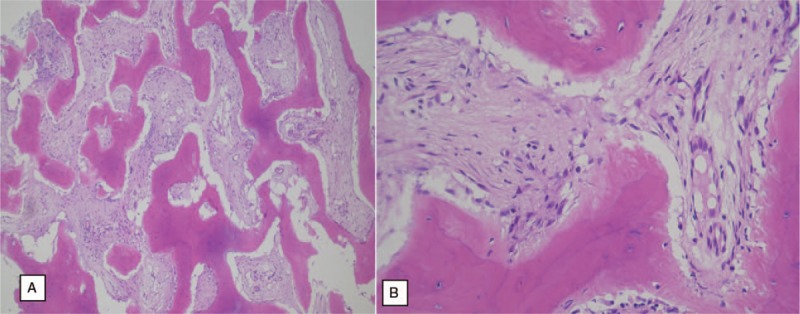
Histologic findings of the excised biopsy revealed “cellular, spindly” stroma and “woven bone without osteoblastic rimming” resembling “Chinese letters” (left 100×, right 400×).

**Figure 5:**
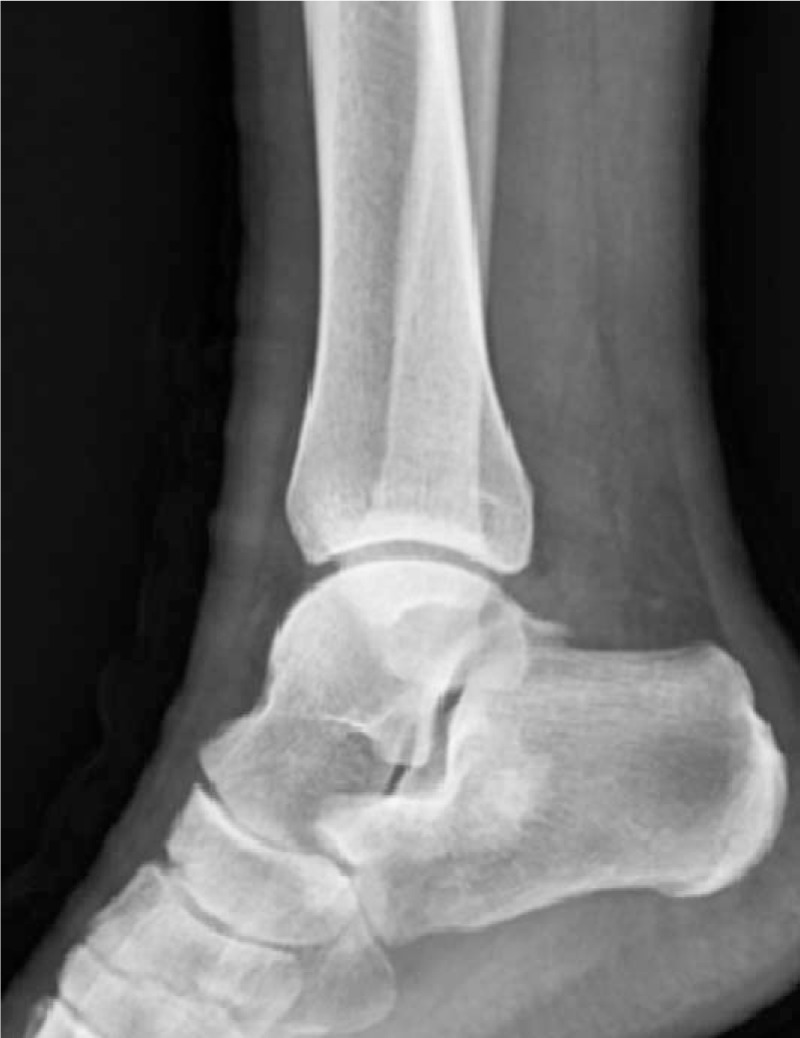
Twelve months postoperatively, a plain radiogram showed a well-consolidated bone graft in the lesions.

A non-enhanced computed tomography (CT) scan showed 5 round-shaped lesions (2.1 cm × 2.3cm, 1.2 cm × 1.5 cm, 0.7 cm × 0.8 cm, 0.5 cm × 0.5 cm and 0.5 cm × 0.4 cm sized), 3 were located in the upper (1 was lateral and 2 were central) and 2 were in the lower medial calcaneus, which showed a sclerotic and osteoblastic lesion under the subtalar joint with a cystic round hole in the calcaneal body. Cortical involvement was developed in the lateral-upper lesion (Fig. 2). Non-enhanced T2-weighted fat suppression magnetic resonance imaging (MRI) showed 5 round-shaped lesions of high signal intensity with mixed low signal intensity. One upper central lesion was septate, the 3 upper lateral lesions were connected, and the medial 2 lesions were separate. Bone marrow edema was noticed under the upper lesions, but there was no soft tissue involvement (Fig. 3). The provisional diagnosis was multiple bone tumors. We performed an excisional biopsy with extended curettage through a direct lateral approach under a C-arm image intensifier and bone grafting with an allogenous bone chip (Cellumed, Seoul, Korea) and autogenous bone marrow aspirate concentrate. Histologic examination of the excised biopsy revealed cellular, spindly stroma and woven bone without osteoblastic rimming resembling Chinese characters indicative of FD (Fig. 4). Twelve months postoperatively, a plain radiogram showed a well-consolidated bone graft in the lesions (Fig. 5).

## 3 Discussion

Monostotic FD commonly develops in the long bones where bone remodeling occurs, such as the proximal femoral shaft (40%), tibia (15%), humerus (5%) and the radius (5%).^[1]^ However, FD in the calcaneus is extremely rare. An extensive English literature search revealed only 3 published case reports on this topic.^[1–3]^

The most common presentation of monostotic FD is a single-bone lesion without any other disturbance.^[4]^ However, the present case showed multiple (5) lesions in the calcaneus. Monostotic FD is known to enlarge in proportion to skeletal growth.^[4]^ Moreover, monostotic FD usually manifests as a single large bone lesion in a long bone, such as the proximal femur, tibia, humerus and radius but the present case showed 5 bone lesions in a short bone, the calcaneus. We believe this phenomenon resulted from the arrest of bone remodeling at different times in the 5 lesions in the calcaneus. The different arrest times in the calcaneus made a boundary between the normal lamellar bone and pathologic woven bone area, unlike previously reported cases that showed enlargement in the same area.^[1–5]^

The common radiologic findings of FD bone lesions generally manifest as cystic, sclerotic or mixed. A typical FD lesion in the axial skeleton appears as an area of radiolucent ground glass matrix, which is usually smooth and homogeneous, not centrally located within the medullary bone. Additionally, FD lesions show sharply demarcated borders and intermediate-to-low signal intensity on T1-weighted image and intermediate-to-high intensity on T2-weighted images.^[6]^ This present case did not show a typical radiolucent ground glass matrix and showed multiple lesions with high signal intensity on T2-weighted image. We provisionally diagnosed the patient with multiple bone tumors, not FD, which needed to be biopsied.

The histopathologic hallmark of FD is fibrous tissue and immature, spindle-shaped, fibroblast-like cells within the bone marrow.^[1]^ These fibrous tissues expand from the medullary cavity to the cortical bone. The strangely-shaped trabeculae have been likened to “alphabet soup” or “Chinese characters.” The histological findings in this case are consistent with typical FD.

Treatment of FD is indicated when it becomes symptomatic or there is a risk of fracture. This patient experienced pain on walking for 2 months that was unresponsive to pain killers. The provisional diagnosis was multiple bone tumors based on the CT and MRI images. An excisional biopsy and bone graft were considered to further evaluate the bone defect. Curettage and bone grafting, with or without bone internal fixation, are generally accepted as the treatment of choice in the majority of cases.^[1–8]^ The choice of grafts, whether allografts or autografts, is controversial. Leet et al concluded that there was no difference in resorption rates between allografts and autografts.^[9]^ They reported that the resorption rate was high, especially at younger ages and in patients with polyostotic disease. The present case was well treated with an allogenous bone graft and autogenous bone marrow aspirate without resorption at the 1-year postoperative follow-up.

## Author contributions

**Conceptualization:** Kwang-Bok Lee.

**Data curation:** Gun-Joo Park, Kwang-Bok Lee.

**Formal analysis:** Jong-Hyun Ko, Kwang-Bok Lee.

**Funding acquisition:** Kwang-Bok Lee.

**Investigation:** Jong-Hyun Ko.

**Methodology:** Gun-Joo Park, Kwang-Bok Lee.

**Software:** Gun-Joo Park.

**Supervision:** Kwang-Bok Lee.

**Writing – original draft:** Jong-Hyun Ko.

**Writing – review & editing:** Kwang-Bok Lee.
